# Effects of Immediate Orthodontic and Orthopedic Forces on Peri-Miniscrew Bones: Histomorphologic and Histomorphometric Assessment in Dogs

**DOI:** 10.1155/2012/851740

**Published:** 2012-12-25

**Authors:** Mansour Rismanchian, Sayed Hamid Raji, Danesh Teimori Rik, Sayed Mohammad Razavi, Navid Khalighinejad, Hossein Goroohi, Hamid Badrian

**Affiliations:** ^1^Department of Prosthodontics, Dental School, Isfahan University of Medical Sciences, Isfahan, Iran; ^2^Department of Orthodontics, Dental School, Isfahan University of Medical Sciences, Isfahan, Iran; ^3^Department of Prosthodontics, Dental School, Zahedan University of Medical Sciences, Isfahan, Iran; ^4^Department of Oral Pathology, Dental School, Isfahan University of Medical Sciences, Isfahan, Iran; ^5^Student of Dentistry, Dental School, Isfahan University of Medical Sciences, Isfahan, Iran

## Abstract

*Aim*. The aim of this study was histomorphologic and histomorphometric evaluation of peri-screw bone after immediate orthodontic and orthopedic forces and comparing them with a control group. *Materials and Methods*. 18 dual-top miniscrews were inserted in the premolar region of three Iranian dogs. Screws were divided into three groups: (1) the first group undergoing immediate orthodontic force of 300 cN, (2) the second group undergoing immediate orthopedic force of 600 cN and (3) a control group. Screws were explanted with adequate amount of surrounding bone after three months. Bone-screw contact (BSC), percentage of lamellar bone, and percentage of woven bone were evaluated. Wilcoxon and Man Whitney tests were used to analyze the data using SPSS software ver. 15 (*α* = 0.05). *Results*. There was no significant difference among the groups in terms of bone-screw contact (*P*  value = 0.42), percentage of lamellar bone (*P*  value = 0.83), and percentage of woven bone (*P*  value = 0.88). *Conclusion*. By applying orthodontic and orthopedic forces to mini-screws the quality of surrounding bone and osseointegration will not be affected. *Clinical Significance*. Application of force to mini-screws is helpful in orthodontic-screw therapies.

## 1. Introduction

Nowadays, a larger number of patients are seeking orthodontic treatments to enhance their quality of life [1]. Since the introduction of orthodontic treatments, anchorage has been a challenge for clinicians [2]. Orthodontic anchorage is defined as resistance to unwanted tooth movement and many ways have been suggested to provide an appropriate anchorage [3]. Traditionally, a large group of teeth or intra- and extraauxiliary devices have been used [2] but some situations like missing teeth, periodontitis, and lack of patients compliance preclude the use of those common ways [4, 5].

For the first time in 1984, Roberts et al. recommended the use of screws as orthodontic anchorage. It was observed that titanium screws remained stable during orthodontic forces, concluding that the use of screws for orthodontic anchorage can be beneficial [4]. Dental screws have some shortcomings that hinder their use. These screws can be placed only in limited sites like retromolar and edentulous areas [6, 7]. Also they impose some troubles such as severity of surgery and long healing time, making them unacceptable for some patients [7]. Due to screws impediments in providing anchorage, miniscrews were introduced by Kanomi. Miniscrews are titanium screws with 1.2 mm diameter and 6.0 mm length [8]. After a while, miniscrews became popular because of their ease of insertion, low cost, and minimal surgical procedures [9–12]. Clinical and experimental studies have evaluated the serviceability of miniscrews concluding that they are highly effective during orthodontic treatments [13]. Despite implants disadvantages in orthodontics, they show higher success rate of 95% in comparison to miniscrews [14]. It has been proposed that the main reason for the lower success rate of miniscrews is biomechanical criteria such as bone quality and the time of loading [15]. When the load is applied prematurely on screws, nonuniform bone-screw contact will be the final result [3, 10]. This phenomenon could be an appropriate consequence for miniscrews, easing their removal after orthodontic treatment period [16]. Recent clinical studies on miniscrews have shown high failure rate in miniscrews used for orthodontic anchorage [17], thus introducing an accurate protocol for biomechanical characteristics of peri-miniscrews bone and a loading protocol seems rational.

Immediate loading can effectively reduce treatment duration which can be beneficial for both clinicians and patients [7]. Researchers have investigated the effect of immediate loads on miniscrews regarding bone-miniscrew contact but none of them surveyed the effect of loading on other aspects of bone reaction [18]. As little information is present regarding the effect of immediate loads on miniscrews and the characteristics of peri-screw bone and as the behavior of peri-miniscrew bone plays an important role in miniscrews stability, the aim of this study was to histomorphologically and histomorphometrically assess the peri-miniscrew bone following the application of immediate orthodontic and orthopedic forces and compare it with the control group.

## 2. Materials and Methods

In this prospective animal study 3 Iranian mixed-breed dogs with almost similar condition (25 kg weight and aged 2 to 3 years old) were selected for the study. All dogs were examined by a veterinarian and declared to be healthy. To prevent probable cross-infection of diseases, all dogs were vaccinated (the vaccines included rabies, influenza, hepatitis, leptospirosis and distemper).

### 2.1. Tooth Extraction and Miniscrew Insertion

Three miniscrews were inserted in the left mandibular and three were inserted in the right maxillary premolar region in each dog.

First, 10 mg ketamine vials were injected intramuscularly, giving general anesthesia. To maintain the anesthesia, Isoflurane (Honeypot lane, London NW, UK) was used. Dextrose saline serum was used to balance electrolytes.

 Periapical radiographies were taken from both jaws. Dogs' mouths were rinsed by chlorhexidine. According to Helsinki rules we were allowed to extract first to fourth premolars. After tooth extraction, 2 cartridges of Lidocaine (Lidocaine, epinephrine 1/100000) were administrated for each quadrant to reduce bleeding. Miniscrews (Dual-top, Anchor screw Jeil Medical, Seoul, Republic of Korea) were sterilized in autoclave. Soft tissues over dogs' alveolar ridge were incised by surgical blade (number 22), because of the thickness of the oral mucosa.

For miniscrew insertion in bone, the appropriate locations of miniscrews were marked using round bur number 1. Then dual-top miniscrews with 1.6 mm diameter and 8 mm length were screwed in the bone using manual screw driver. To make sure that the bone around each screw would not interfere with the bone around adjacent screw, 20 mm distance was considered between miniscrews. To prevent overheating of the surgical site, the region was rinsed with physiologic serum. Miniscrews were placed perpendicular to the bone surface, parallel to each other. Miniscrews were screwed until their last threads.

After miniscrew insertion, in cases where the thick soft tissue was assumed to overerupt on the miniscrews surface, an orthodontic separator was used to retract soft tissue. The separators were extracted after the healing of the soft tissue.

### 2.2. Orthodontic and Orthopedic Force Application

**Figure 1:**
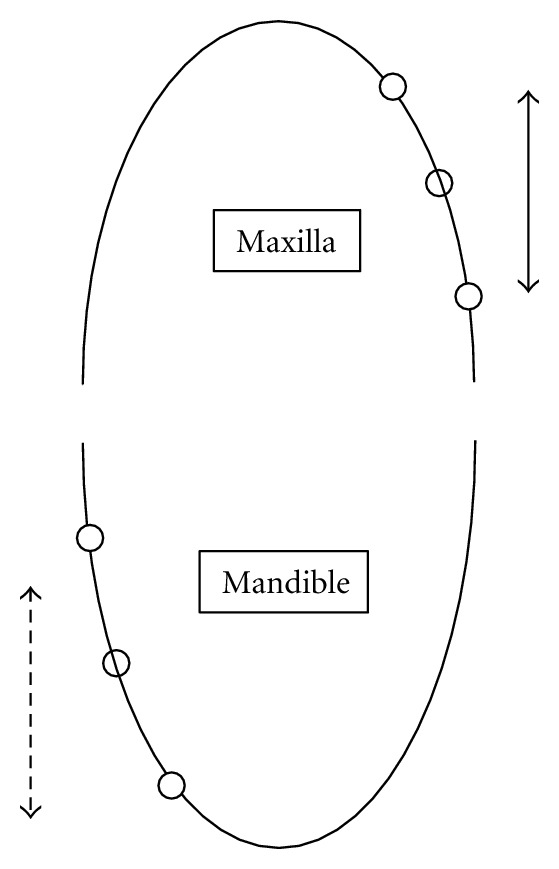
Schematic scheme of miniscrew sites. The distance between adjacent miniscrews is 20 mm. White circles stand for screws undergoing orthodontic force. Black circles stand for control screws. 300 cN was applied in maxilla (arrow A). 600 cN force was applied in mandible (arrow B).

In each dog, two miniscrews underwent 300 cN orthodontic force, two underwent 600 cN orthopedic force and two miniscrews were considered as control (Figure 1). Immediately after placement of the miniscrews, Ni-Ti coil springs (Sentalloy, GAC, Central Islip, N.Y. USA) were attached to the upper part of 6 miniscrews (two in each dog) by means of .012′′ ligature wires, in the way that the springs were stretched 20 mm (to produce 300 cN force).

600 cN force was applied to other 6 miniscrews. Since appropriate Ni-Ti coil springs applying 600 cN force were not available, 2 springs similar to those with 300 cN force were stretched 20 and used simultaneously.

Last 6 miniscrews were considered as control group and were not loaded.

### 2.3. Postoperative Care

After the operation, the dogs received an intramuscular ampoule of penicillin G benzathine/procaine sodium 2 : 1 : 1 and a neutralized sulfonamide (Pentazole) 1 cc/kg. To keep dog's oral health in an optimum condition, 0.12% chlorhexidine mouth rinse and brushes were used weekly. After the operation, the dogs were fed with cooked ground chicken once a day.

### 2.4. Followup

Six weeks after surgery, following anaesthetizing the dogs, their mouths were rinsed using brush and chlorhexidine, springs were detached, and their force was rechecked. The springs were attached again. It should be mentioned that the springs were replaced only if deformation occurred.

### 2.5. Extracting the Specimens

Twelve weeks after surgery, miniscrews and springs were cleaned using chlorhexidine and brush and the forces were checked and followed by removing the springs. Trephine burs with 6 mm diameter were used to cut out miniscrews with adequate amount of bone, 12 weeks after surgery.

### 2.6. Histological Preparation

Extracted specimens were kept in a glutaraldehyde solution for 6 hours. Embedded in series of graded alcohol, they were dehydrated and mounted precisely in a block of self-cured transparent acrylic resin (Meliodent; Heraeuskulzer, Berkshire, UK). Ground sections were then prepared, using Microtome (Accutom-50, Stuers, Copenhagen, Denmark). Sections were made along screws' longitudinal axis and in mesiodistal dimension with approximate 250–350 *μ*m thickness. The specimens were then thinned to 100–150 *μ*m using an abrasive. All the specimens were stained by Masson's trichrome method. After staining, the specimens were investigated under optical microscope (×100 magnification) (Ziess Germany). The amount of BSC and percentage of different types of bones (lamellar and woven) were recorded at the tension side and the pressure side, using calibrated lens. The percentage of lamellar and woven bone within 2 mm around miniscrews was determined by counting the number of the lens's cells including the specific type of bone. Woven bone is characterized by cellular structure with collagen fibers spreading in different directions irregularly. Lamellar bone is known with its lamellate and lamellar structure with lower cellularity compared to woven bone, while the collagen fibers spread more regularly. The sections of specimens were also observed by Adobe Photoshop 7.0 (San Jose, CA) and the amount of BSC and percentage of lamellar and woven bone were recorded to confirm data.

### 2.7. Ethical Considerations

The present study was approved by the Ethics Committee of Dental School of Isfahan. The votum of committee is 386260. In this study dogs were not sacrificed after the procedures.

### 2.8. Statistical Analysis

Wilcoxon and Mann Whitney tests were used to analyze the data using SPSS software ver. 15 (*α* = 0.05).

## 3. Results

One of the miniscrews with 300 cN force was excluded from the study because of getting loose when the dog bit the fence bars of the cage, during the follow-up period. The area exhibited no signs of inflammation. Therefore 5 miniscrews with 300 cN force, 6 of them with 600 cN force and 6 control miniscrews, remained for data analysis.

In the present study, total bone-miniscrew contact, total woven and lamellar bones were investigated separately at the tension and pressure sites. No connective tissues were observed around the implants.

## 4. Bone-Miniscrew Contact (BMSC)

**Figure 2:**
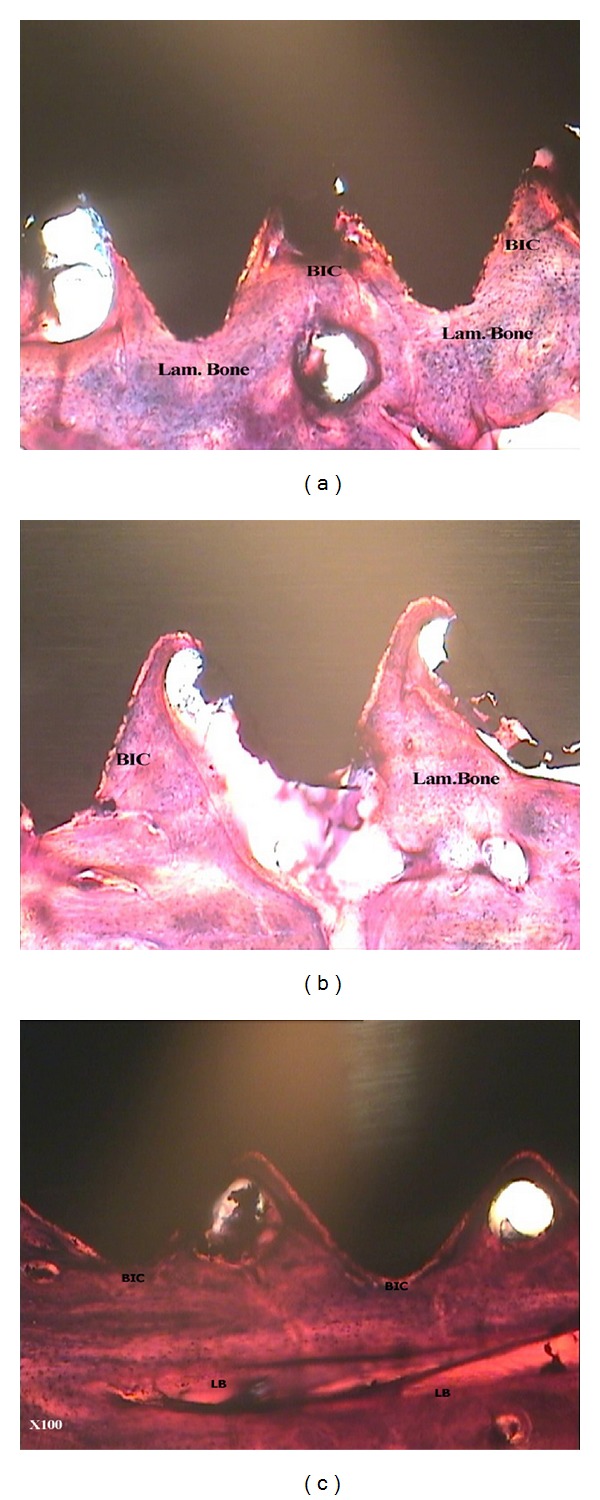
Photomicrographs of bone-to-miniscrew contact in different groups: (a) immediate orthodontic group, (b) immediate orthopedic group, and (c) control group. Note the quality of bone formation (woven and lamellar) around screws. Masson's trichrome; original magnification ×100 (BSC: Bone-to-miniscrew contact, L.B: Lamellar bone, W.B: woven bone).

Mean percent of total BMSC was 73.53 ± 14.505, 71.025 ± 13.253, and 64.641 ± 4.590 for 300 cN group, 600 cN, and control group, respectively (Figure 2). The mean values of total bone miniscrew contact and bone miniscrew contact at tension side and pressure side were not significantly different among groups (300 cN, 600 cN, and 0 cN) (*P*  value > 0.05) as illustrated in Table 1.

## 5. Peri-Miniscrews Lamellar Bone (LB)

Mean percentage of total LB within 2 mm around miniscrews was 59.3 ± 2.996, 59.241 ± 2.610, and 58.316 ± 3.639 and for 300 cN group, 600 cN group, and control group respectively. The mean values of peri-miniscrew lamellar bone were not significantly different among the groups (300 cN, 600 cN, and 0 cN) in both tension and pressure sides (*P*  value > 0.05) as illustrated in Table 2.

## 6. Peri-Miniscrews Woven Bone (WB)

Mean percentage of total peri-screw woven bone within 2 mm around miniscrew for control group, 300 cN group, and 600 cN group was 33.025 ± 2.53845, 33.65 ± 1.816, and 33.33 ± 1.802, respectively. The mean values of peri-miniscrew woven bone were not significant in groups (300 cN, 600 cN, and 0 cN) in both tension and pressure sides (*P*  value > 0.05). More details are illustrated in Table 3.

## 7. Discussion

The success of early loading in miniscrews is highly dependent on the primary stability [19, 20] and the primary stability depends mainly on the peri-screw bone characteristics [21–23]. Immediate loading of miniscrews has been suggested in some reports [9, 10, 24, 25], but there are not much supporting histological data [18], so in this study a number of criteria including bone-screw contact (BSC), and lamellar and woven bone formation around miniscrews were investigated.

Except one of the miniscrews in 300 cN groups that was excluded due to traumatic injuries, none of the miniscrews got lost during the study. This finding indicates that immediate loads on miniscrews do no compromise miniscrews condition and cannot be a reason of anchorage loss during orthodontic treatments. This finding confirmed Lee CY's study who declared that immediate loads on miniscrews can effectively reduce the treatment duration with appropriate consequences [26].

In the present study, there was a higher percentage of BSC in the loaded groups in comparison to unloaded group although the difference was not statistically significant. Freire et al. applied 250 gram forces on miniscrews immediately, after one week, and after three weeks. This study showed that immediate loads on miniscrews can increase bone screw contact and these results are in agreement with the result of the present study [27]. So it seems that immediate orthodontic forces may provide an appropriate condition for miniscrews to act as an orthodontics anchorage.

The results of the present study are in agreement with Deguchi et al. [28] and Ohmae et al. [29]. They found no significant differences between the amount of BSC in the loaded group and the unloaded control screws despite higher BSC in loaded groups in Beagle dogs.

Also in the present study, there was no significant difference between mean BSC in tension side and pressure side. This result confirmed the results of Cesareluzi study that showed there is no significant difference in the BSC and bone healing pattern in pressure side and tension side. It may suggest that the application of 300 and 600 cN forces does not have adverse effects on pre-miniscrew bone.

The peri-miniscrew bone reaction regarding woven bone and lamellar bone formation was investigated in the present study. None of the samples exhibited a significant difference in woven or lamellar bone formation around miniscrews. This finding indicated that immediate orthodontic forces will not probably jeopardize miniscrews histomorphometric and histomorphological condition during comprehensive treatments.

It should be emphasized that BSC is a time-independent interaction and orthodontic treatments are usually long-term treatments. On the other hand, a dependable anchorage unit should not move during orthodontic treatments. Further studies should be done to investigate the effect of immediate loads in different time intervals.

## 8. Conclusions

It can be concluded that application of orthodontic and orthopedic immediate forces on miniscrews will not histomorphometrically or histomorphologically affect the surrounding bone. Immediate forces do not have any negative effect on BSC and amount of lamellar or woven bone around miniscrews.

## Figures and Tables

**Table 1:** Statistical indices of total bone-mini-screw contact, at tension side and pressure side of 300 cN, 600 cN, and control group (0 cN).

BSC	Group (cN)	Number	Mean ± SD	*P* value
Total	300	5	73.53 ± 14.50	0.426
	600	6	71.02 ± 13.2	
	0	6	64.64 ± 4.59	
Pressure side	300	5	74.00 ± 15.80	0.473
	600	6	71.08 ± 14.41	
	0	6	64.95 ± 3.98	
Tension side	300	5	73.06 ± 13.46	0.385
	600	6	71.05 ± 12.04	
	0	6	64.33 ± 5.896	

**Table 2:** Statistical indices for lamellar bone around mini-screw within 2 mm at pressure side and at tension side.

BSC	Group (cN)	Number	Mean ± SD	*P* value
Total	300	5	59.30 ± 2.99	0.836
	600	6	59.24 ± 2.61	
	0	6	58.31 ± 3.63	

Pressure side	300	5	59.50 ± 3.64	0.406
	600	6	59.95 ± 2.5	
	0	6	57.46 ± 3.606	

Tension side	300	5	59.10 ± 2.50	0.842
	600	6	58.53 ± 2.86	
	0	6	58.08 ± 3.07	

**Table 3:** Statistical indices for woven bone around mini-screw within 2 mm at pressure side and at tension side.

BSC	Group (cN)	Number	Mean ± SD	*P* value
Total	300	5	33.65 ± 1.81	0.887
	600	6	33.33 ± 1.80	
	0	6	33.02 ± 2.53	

Pressure side	300	5	33.80 ± 1.78	0.372
	600	6	32.50 ± 1.04	
	0	6	34.05 ± 2.67	

Tension side	300	5	33.50 ± 2.06	0.720
	600	6	34.16 ± 2.67	
	0	6	33.08 ± 2.08	
